# Fitness costs associated with building and maintaining the burying beetle’s carrion nest

**DOI:** 10.1038/srep35293

**Published:** 2016-10-13

**Authors:** Ornela De Gasperin, Ana Duarte, Jolyon Troscianko, Rebecca M. Kilner

**Affiliations:** 1 Department of Zoology, University of Cambridge, CB2 3EJ, UK; 2 Department of Ecology and Evolution, University of Lausanne, 1015, Lausanne, Switzerland; 3 Centre for Ecology and Conservation, University of Exeter, Penryn Campus, TR10 9FE, UK

## Abstract

It is well-known that features of animal nest architecture can be explained by fitness benefits gained by the offspring housed within. Here we focus on the little-tested suggestion that the fitness costs associated with building and maintaining a nest should additionally account for aspects of its architecture. Burying beetles prepare an edible nest for their young from a small vertebrate carcass, by ripping off any fur or feathers and rolling the flesh into a rounded ball. We found evidence that only larger beetles are able to construct rounder carcass nests, and that rounder carcass nests are associated with lower maintenance costs. Offspring success, however, was not explained by nest roundness. Our experiment thus provides rare support for the suggestion that construction and maintenance costs are key to understanding animal architecture.

Nest-building is widespread among animals, and the extraordinary diversity in nest architecture is thought to be adaptive^1^. Specifically, natural selection is predicted to favour designs that enhance reproductive success while minimising building and maintenance costs to parental fitness^1^,^2^,^3^. Although it is clear how some aspects of nest architecture function to promote successful reproduction^1^,^2^,^3^ it has been much harder to isolate elements of nest design that are linked to the costs borne by parents of nest construction and maintenance.

Just as with other behavioural traits^4^, the costs associated specifically with nest-building can be exposed in different ways. One approach is to seek a correlation with parental quality. If constructing a nest is entirely cost-free, then all parents should be able to construct an ‘ideal’ nest, irrespective of their quality. If there are costs involved, however, only the highest quality parents should be able to build this ideal nest. A different technique for quantifying costs is to measure an individual’s residual fitness after nest construction or maintenance: the lower its residual fitness, the greater the cost it has incurred through its nesting activity. The advantage of each of these approaches is that the costs of nest-building can be estimated without quantifying details of the behaviour involved (see also^5^). Here we use these methods to determine whether the costs of nest-building and maintenance are correlated with natural variation in burying beetle nest architecture.

Burying beetles (*Nicrophorus vespilloides*) prepare the carcass of a small vertebrate for reproduction by removing any fur or feathers. They roll the flesh into a ball by sinking it below ground and repeatedly thrusting it against a tunnel wall, turning it between thrusts^6^. Within two days they transform the carcass into a naked ball of flesh. This becomes an edible nest, which nourishes and houses the developing larvae^7^. Beetles also defend carcasses from microbial decay by smearing them with antimicrobial exudates^8^,^9^ and this imposes a fitness cost on the parents^10^. We investigated the costs of building and maintaining this spherical carcass nest, in relation to any benefits that might be gained by the larvae from its spherical nature or ‘sphericity’.

A perfect sphere of flesh creates the lowest possible surface area to volume ratio of the carcass, which could have several associated advantages for the parents. First, it reduces the area to be defended against microbial competitors and hence minimizes the costs of producing antimicrobial exudates, relative to the amount of resources on the carcass. Second, male and female burying beetles typically patrol the carcass surface in search of fly larvae, which compete with beetles for resources on the carcass^6^,^11^,^12^. A rounder carcass may minimise the time spent patrolling the carcass surface, for a given carcass size. Third, to determine their brood size, burying beetles assess carcass volume rather than carcass mass^13^ and this they might achieve by measuring the time to walk around the carcass (this is how some parasitoids (e.g. *Trichogramma* spp) assess the size of their hosts to determine clutch size^14^). A rounder carcass could therefore provide beetles with more accurate information about carcass volume, which could in turn reduce variability in brood size or larval mass. Finally, a spherical carcass nest could be advantageous from the brood’s perspective too because it could slow the rate of carcass desiccation^15^, making it easier for larvae to feed on the flesh^6^.

The sphericity of the carcass nest is naturally highly variable (personal observation; Fig. 1A). We investigated whether this variation is associated with the potential costs of nest construction, the costs of nest maintenance, and aspects of offspring performance.

We had four predictions: (1) larger parents should produce rounder carcass nests because they can more effectively manoeuvre the carcass to roll it up; (2) rounder carcass nests should minimise the costs of nest maintenance^10^, which in turn should increase residual parental fitness; (3) rounder carcass nests should induce lower variance in clutch size and brood size, because carcass volume can more accurately be estimated by parents; and (4) rounder carcass nests should be associated with greater brood size and larval mass, either because rounder nests dry out more slowly, making it easier for the offspring to consume them, or because they are better defended against microbes.

## Methods

### General Methods

The beetles used in this experiment came from a laboratory population founded in 2005 and kept in the Department of Zoology, at the University of Cambridge. Field-caught beetles were introduced annually to prevent inbreeding. Individuals were kept in small plastic containers (12 cm × 8 cm × 2 cm) filled with moist soil. We maintained the colony at 20 °C and on a 16:8 light to dark cycle and fed individuals twice per week with minced beef.

## Experiment

We have described other data collected from this experiment in detail elsewhere^16^ (experiment 1), but the analyses presented here are novel and focus only on individuals not exposed to mites. Briefly, we bred unrelated 2–3 week old virgin adults using a container that allowed parents to leave the carcass nest at a time of their choosing, just as they would in nature^16^. The breeding box was divided in two with a cardboard partition. One side was filled two thirds with soil, and held the beetles and mouse. The other side had a shallow layer of soil. In the centre of the partition was a small plastic tube. Beetles could leave through this tube but a fabric tunnel on the other side prevented them from returning (see Fig. 1 in ref. 16). Pairs were given a mouse carcass weighing 8–15 g (mean = 11.95; SD = 1.82). We checked the boxes every 2 h, from 08:00 to 20:00, and collected any beetle that had abandoned the nest and recorded the time of departure. Eight days after pairing (when larvae disperse from the carcass) we counted and weighed the brood, and kept the parents. We fed the adults twice per week with minced beef until they died, recorded their lifespan to estimate residual fitness and measured their pronotum width to estimate their size. Lifespan is sometimes considered a poor measure of residual fitness. However, as we explain in detail elsewhere^17^ the burying beetle’s unusual natural history, and in particular the opportunistic nature of its reproduction, mean that lifespan and fecundity are tightly connected in this species – as we have demonstrated in previous experiments^10^. We performed this experiment in two batches, yielding 45 successful breeding pairs. All experiments complied with the ethics regulations at the University of Cambridge.

### Measuring carcass roundness

**Figure 1:**
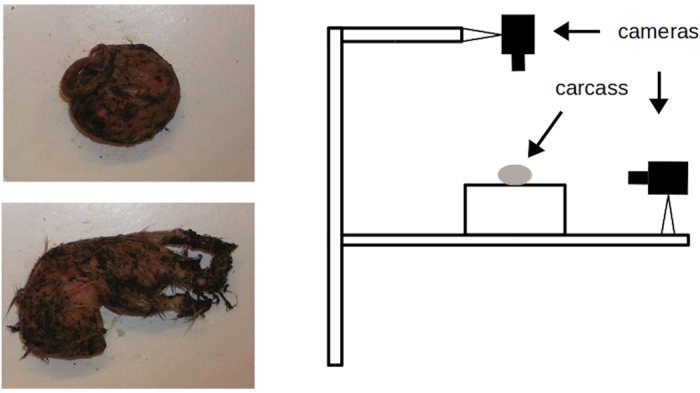
Left: Two prepared carcass nests (photographed from above), to illustrate variation in nest roundness. Right: Diagram illustrating how the pictures were taken.

Fifty-six hours after pairing we photographed each prepared carcass against a white background from the side and top, using two identical cameras (model: Fujifilm av200), each placed 30 cm away from the carcass (Fig. 1B). We also weighed the carcasses again to estimate the extent of carrion consumed by parents during carcass preparation, prior to larval hatching. Carcasses were then returned to their owners.

We used the carcass images to calculate the ‘roundness’ of each carcass, using roundness as a two-dimensional proxy for carcass sphericity. We estimated carcass roundness using a custom written script in ImageJ (version 1.46), which can be found in the supplementary materials. The script selected the blue channels from the top and side photos as these provided the highest contrast between the nests and their white background. The images were thresholded to separate the nests from their backgrounds, and a median filter of 25 pixels was used to remove the smallest details (i.e. hair or soil smaller than 1 mm across – the photographs were 27.1 and 27.6 pixels per mm from the top and side images, respectively). We assumed that if the carcass was perfectly spherical then each image should be a circle, and the perimeter of that circle should be the same as the perimeter of the carcass image. We calculated the roundness of each image as the ratio of the area covered by the flesh ball to the area that a perfect circle of the same perimeter length (= circumference) would cover, such that a score of 1 denotes a perfect circle. To derive an overall roundness score for each carcass, we took the average of the measures from both images. Due to their confounding influence on roundness measures, tails, legs and large pieces of soil on the carcasses were removed prior to processing with white circles using GIMP (version 2.6.11). Throughout the process of measuring roundness, the experimenter had no knowledge of key correlates in the beetles such as size or residual fitness.

## Statistical analysis

We analysed the data using R^18^. All our response variables were normally distributed: Shapiro test for male lifespan: W = 0.97; *P* = 0.31; for female lifespan: W = 0.97; *P* = 0.31; for brood size: W = 0.97; *P* = 0.43; for average larval mass: W = 0.97; *P* = 0.34; and for carcass sphericity: W = 0.98; *P* = 0.64. We first used generalized linear mixed models with normal distribution of error and included the block as a random effect in each model (lme4 package^19^). However, after evaluating the models we removed the random effect as the block explained almost no variance, and used linear models instead (lm function). We reduced each model with backward elimination using the AIC^20^, obtained *p-*values with the summary function and checked the distribution of the residuals from the final models.

Prediction 1: Do larger parents prepare rounder carcasses?

We analysed the extent to which carcass roundness could be explained by variation in male and female size. This analysis also controlled for the potentially confounding effects of the mass of the unprepared carcass, and the desertion time of the males and females, by including them as covariates in the model.

Prediction 2: Do rounder carcasses minimise maintenance costs?

We quantified the extent to which male or female lifespan could be explained by variation in the roundness of the carcass. In these analyses we controlled for the size of each parent, the mass of the carcass (either prepared or unprepared, or the change in mass between unprepared and prepared carcasses), and the desertion time of the male and female, by including them as covariates.

Prediction 3: Do rounder carcasses reduce variance in brood size and larval mass?

We split the data set according to whether the carcasses were ‘round’ (defined as above mean roundness), or ‘not round’ (below mean roundness). Using Levene’s test for homogeneity of variance, we compared the variance of the following traits on ‘round’ and ‘not round’ carcasses: a) the size of the brood, b) the average larval mass, and c) the larval density (calculated as brood size/carcass mass^21^).

Prediction 4: Do rounder carcasses promote offspring performance?

We quantified the extent to which the size or mass of the brood, or the average larval mass (obtained by dividing brood mass by brood size), could be explained by variation in carcass roundness. Because brood size and brood mass were highly correlated (Pearson *r* = 0.93, *p* < 0.00001) we only considered brood size in our analysis. In these analyses we again controlled for the size of each parent, the mass of the carcass (either prepared or unprepared, or the change in mass between unprepared and prepared carcasses), and the desertion time of the male and female beetles, by including them as covariates. In the analysis of average larval mass we also controlled for brood size.

## Results

Prediction 1: Do larger parents prepare rounder carcasses?

**Figure 2:**
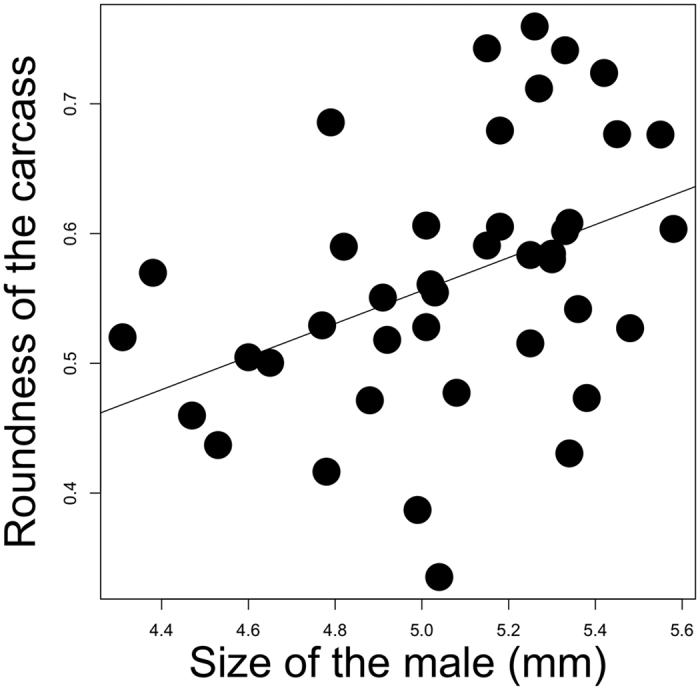
The relationship between the size of the male and the roundness of his carcass nest. The graph shows the linear regression between the raw values.

Male size was positively correlated with carcass roundness (Table 1; Fig. 2), but female size was not (*p* = 0.097). Neither the desertion time of the male (*p* = 0.19), nor of the female (*p* = 0.66), nor the mass of the unprepared carcass (*p* = 0.92), nor the loss in carcass mass during its preparation (*p* = 0.98) explained variation in the roundness of the prepared carcass.

Prediction 2: Do rounder carcasses minimise maintenance costs?

**Figure 3:**
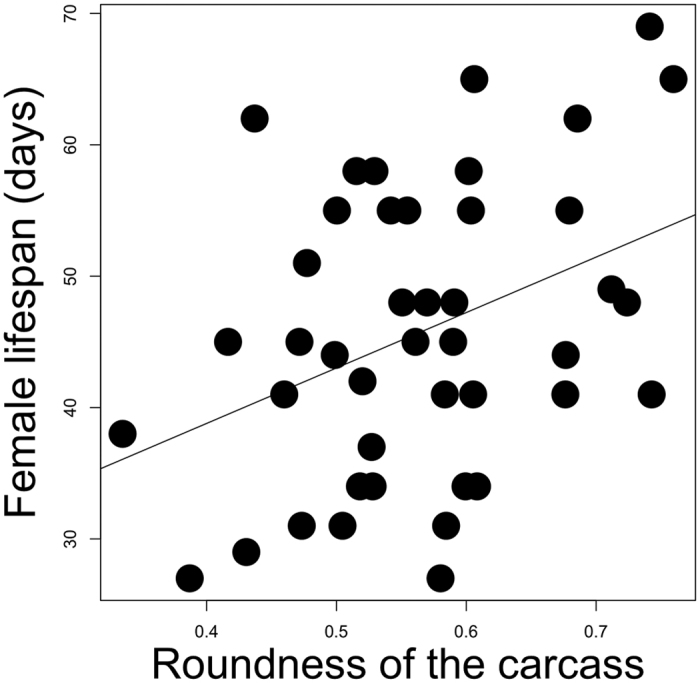
The relationship between the lifespan of the female and the roundness of her carcass nest. The graph shows the linear regression between the raw values.

The roundness of the prepared carcass positively predicted female lifespan (Table 1; Fig. 3) but not male lifespan (*p* = 0.22). Neither the size of the male (*p* = 0.63), nor of the female (*p* = 0.33), nor the desertion time of the male (*p* = 0.40), nor of the female (*p* = 0.21), explained variation in female lifespan.

Prediction 3: Do rounder carcasses reduce variance in brood size and larval mass?

The roundness of the carcass nest did not affect variance in average larval mass (*F*_43,1_ = 0.13; *p* = 0.71), nor variance in brood size (*F*_43,1_ = 0.003; *p* = 0.95), nor variance in the larval density (*F*_43,1_ = 0.25; *p* = 0.61).

Prediction 4: Do rounder carcasses promote offspring success?

Mean ± S. E. M. of the size of the brood = 16 ± 1.30 (number of larvae); mean ± S. E. M of the average larval mass = 0.12 ± 0.004 (g); and mean ± S. E. M of the mass of the brood = 1.86 ± 0.11 (g). Carcass roundness did not explain variation in brood size (*p* = 0.12), where there was a non-significant trend for rounder carcasses to yield smaller broods, after controlling for other factors in the model (Estimate = −20.75; SE = 13.37); nor did it explain average larval mass (*p* = 0.42), though there was a very weak trend for rounder carcasses to yield larger offspring after controlling for other factors in the model (Estimate = 1.88e − 02; SE = 2.34e − 02). Offspring performance was instead associated with the mass of the carcass (Table 1).

## Discussion

Our analyses show that: (1) larger males produce rounder carcass nests; (2) females with rounder carcass nests live longer after reproduction; but that (3) rounder carcass nests are not associated with measures of offspring success, nor with the variance in offspring performance.

We infer from the first result that the roundness of the carcass reflects a cost of rounding the unwieldy flesh of a carcass into a ball, which only larger individuals can bear. Our analyses further suggest that males do more rolling of the carcass flesh than females. This could be tested directly in future work. Surprisingly, we found no relationship between carcass roundness and carcass mass. It might be thought that larger carcasses are more difficult to ball-up, whereas small carcasses are easier to manoeuvre, even by small-sized beetles. It is possible that we did not find evidence to support this possibility because we deliberately minimized variation in carcass size, as part of our experimental design.

The second result is consistent with the prediction that a more spherical carcass nest incurs a lower maintenance cost. Carcass maintenance includes making and applying antimicrobial anal exudates, and patrolling the carcass for fly eggs. Of these two activities, we know that females contribute more to antimicrobial maintenance activities^8^,^22^, and this may explain why only female residual fitness was correlated with carcass roundness while male residual fitness was not. This interpretation of the data implies that males drive the correlation between carcass roundness and residual female fitness. Thus, males unilaterally make a rounder carcass, and females then benefit accordingly from the lower associated costs of carcass maintenance. But we could equally plausibly reverse the causal arrow and argue that females drive the association between carcass roundness and their residual fitness. Perhaps males assess the quality of their female partner before carcass preparation and differentially allocate greater effort into making a rounder carcass when the female is of higher quality (where ‘quality’ is defined in relation to the costs sustained from the duties of parental care^17^). Further work is required to determine the direction of causality in this correlative result.

Other interpretations of this result are possible but, we contend, are more improbable. For example, perhaps rounder carcasses are sculpted when females consume more of the carcass nest themselves during its preparation, and this offsets their costs of reproduction (cf^23^). However, there was no relationship between the change in carcass mass and carcass roundness as predicted by this scenario. Alternatively, perhaps high quality females contribute more to making a rounder nest, and coincidentally live longer after reproduction. However, our results do not support this hypothesis either because female size had a marginally negative effect on carcass roundness.

Our third finding was that measures of offspring performance were better explained by the mass of the prepared carcass nest, rather than by our measure of nest roundness. We also found no evidence that a rounder carcass yielded less variation in brood size or larval size. This does not mean that a round carcass is of no potential benefit to offspring at all – just that these benefits were harder to detect than the costs to parents associated with building and maintaining a round carcass nest. It is possible, for example, that any benefits that larvae derived from being raised on a rounder carcass were concealed by the correlations between male size and carcass roundness, and between male size and larval mass (Table 1). Teasing apart the separate contributions of male size and carcass roundness to larval mass at dispersal will require a cross-fostering experiment.

If it transpires from these results that a rounder carcass still yields no measureable benefits to offspring then we are left with the puzzle of understanding why the male pays the cost of producing a rounder carcass, seemingly for no personal fitness benefit. One possibility is the larvae benefit from a rounder carcass in ways we have not measured – perhaps because under more natural conditions, a rounder carcass is less vulnerable to attack by rival conspecifics or rival microbes. Or it may be that the cost of making a rounder carcass is correlated with some other component of carcass preparation, such as speed or depth of burial, and this component contributes more directly to larval fitness. Finally, it may be that the male gains fitness through the increased longevity that his sexual partner enjoys as a result of breeding on a rounder carcass, and may sire future offspring with sperm she has stored. However, this seems unlikely given the high levels of promiscuity in *N. vespilloides* and that the last mate typically gains most paternity of the brood^24^.

In summary, and based on the strength of the correlations we report here, we suggest that the roundness of the carcass nest is more strongly associated with the construction and maintenance costs for parents than with the benefits derived by offspring. Our experiment therefore provides rare confirmation of the theoretical supposition^2^ that the architecture of animal nests can be explained by construction and maintenance costs to parents, in addition to any potential benefits gained by the offspring housed within.

## Additional Information

**How to cite this article**: De Gasperin, O. *et al*. Fitness costs associated with building and maintaining the burying beetle’s carrion nest. *Sci. Rep.* **6**, 35293; doi: 10.1038/srep35293 (2016).

## Supplementary Material

Supplementary Information

Supplementary Dataset 1

## Figures and Tables

**Table 1:** Minimal adequate models for each response variable.

Factor	Estimate	SE	*t*	*p*
**Roundness of the prepared carcass**				
Male size	0.10	0.04	2.37	**0.02**
Female size	−0.08	0.05	−1.7	0.09
**Female lifespan**				
Change in mass between unprepared and prepared carcasses	4.80	2.50	1.91	0.06
Carcass roundness	43.52	15.78	2.77	**0.008**
**Male lifespan**				
Mass of the prepared carcass	3.45	1.27	2.87	**0.006**
**Brood size**				
Mass of the prepared carcass	1.89	0.87	2.16	**0.03**
Male size	9.35	3.99	2.34	**0.02**
**Average larval mass**				
Mass of the prepared carcass	0.006	0.001	4.42	**<0.00001**
Brood size	−0.002	0.002	−9.27	**<0.00001**
Male size	0.001	0.006	1.85	0.07
Female desertion time	0.0004	0.00007	6.09	**<0.00001**

*P* < 0.05 values are shown in bold.
