# Supplementary material for: Fitness costs associated with building and maintaining the burying beetle’s carrion nest
Source: Sci Rep. 2016 Oct 13;6:35293. doi: 10.1038/srep35293 (PMC5062497; doi:10.1038/srep35293)
Supplement: Supplementary Information [file srep35293-s1.pdf]

Fitness costs associated with building and maintaining the burying beetle's carrion nest

Ornela De Gasperin[1-2*], Ana Duarte[1] Jolyon Troscianko[3] and Rebecca M. Kilner[1]

[1]Department of Zoology, University of Cambridge, CB2 3EJ, UK

[2] Department of Ecology and Evolution, University of Lausanne, 1015, Lausanne, Switzerland

[3]Centre for Ecology and Conservation, University of Exeter, Penryn Campus, TR10 9FE, UK

*Ornela De Gasperin;

ornela.gasperin@gmail.com;

Tel: +41 21 692 42 45;

Fax: +41 21 692 26 15

//	Title: Mouse ball surface area & volume calculator

//	Author: Jolyon Troscianko

//	Date: 30/04/2012

//

//

//	Description:

//	A photo of a mouse ball must be taken from above and from the side.

//	Photos must be taken from as close to 90 degrees apart as possible.

//	The script uses thresholding (which can be manually adjusted) to separate the mouse ball from the background.

//	Therefore the background should be white, and contrast the black mouse ball. The blue channel is used as this is the darkest

//	The background must be clean - any black dots/dirt can confuse the calculations

//	The script then uses a median filter to remove unwanted artefacts/noise/dirt etc... this can be adjusted

//	Next the number of pixels in each vertical slice across the mouse ball is counted, and the nearest slice in the orthogonal picture is found and counted

//	These are converted to millimeters in height and width at each pixel slice through the mouse

//	The circumfrence of an elipse is approximated from width and height using C = PI * (a+b) * (1+(3*pow((a-b)/(a+b),2) / (10+pow(4-(3*pow((a-b)/(a+b),2)),0.5))))

//	The area of each elipse is calculated as A = PI * a * b

//	The volume of each slice is calcualted from the known pixels per millimeter, and summed across all slices

//	The area is the sum of the elliptical prism of each slice, plus the end of each surface

//	This method overestimates surface area due to the steps being counted on both vertical and end surfaces, but this is a systematic overestimation, so relative statistics shouldn't be affected

//

//	Update: now measures sphericity to avoid above problem of overestimating surface area.

//	Sphericity is taken as the average difference between the area of a circle with a circumference equal to the observed perimeter length of the mouse ball, and the observed area of the mouse ball

//	A sphericity value of 1 would be a perfect circle, lower values indicate worsenning sphericity.

//	topPath=File.openDialog("Select top-down image"); // get file location

//	sidePath=File.openDialog("Select side image");	// get file location

```
	Dialog.create("Settings");
		Dialog.addMessage("Pixel/distance calibration:");
		Dialog.addNumber("Top-down (pixels per mm)", 27.0636);
		Dialog.addNumber("Side (pixels per mm)", 27.6605);
		Dialog.addMessage("\nImage processing:");
		Dialog.addNumber("Threshold", 70);
		Dialog.addNumber("Smoothing radius (pixels)", 25);
		Dialog.addMessage("\nCamera positioning:");
		Dialog.addCheckbox("Flip image", true);
	Dialog.show();

	topPixelsMm = Dialog.getNumber();
	sidePixelsMm = Dialog.getNumber();;
	thresholdVal = Dialog.getNumber();;;
	smoothingVal= Dialog.getNumber();;;;
	flip = Dialog.getCheckbox();

// FILE LOCATIONS

	topDir=getDirectory("Directory containing top-down images");		// select working directory
	topFileList=getFileList(topDir);					// list of images in directory

	sideDir=getDirectory("Directory containing side-on images");		// select working directory
	sideFileList=getFileList(sideDir);					// list of images in directory

	if(topFileList.length > sideFileList.length)	// error if there are unequal numbers of files
		exit("Error: unequal number of files in selected folders");

	if(topFileList.length < sideFileList.length)
		exit("Error: unequal number of files in selected folders");

print("\n________________________________");
```

```
print("SETTINGS");
print("     Threshold: " + thresholdVal);
print("     Smoothing Radius: " + smoothingVal);


// START OF BATCH PROCESSING LOOP


for(z=0; z<topFileList.length; z++1){
        topPath = topDir+topFileList[z];
        sidePath = sideDir+sideFileList[z];



// OPEN & PROCESS TOP IMAGE


        open(topPath);
        run("RGB Stack");
        run("Delete Slice");          // only uses the blue channel (channel with highest contrast)
        run("Delete Slice");
        //setTool(0);        //Rectangle tool
        //waitForUser("Select the Target", "Draw a box to cover the mouse ball\nensure no other dark objects are
selected");


        //run("Crop");


        if(flip==1);          // if ticked, flips image so that both are the same way round
        run("Flip Horizontally");


        setThreshold(0, thresholdVal);
        run("Convert to Mask");


        medianScript = "radius="+smoothingVal+" slice";
        run("Median...", medianScript);      // threshold
```

```
    w = getWidth();  // image dimensions
    h = getHeight();

// SELECT CENTRE

    doWand(w/2, h/2, 1, "4-connected");

    List.setMeasurements;      // save measurements of perimeter & area
    topPerim = List.getValue("Perim.");
    topArea = List.getValue("Area");

    //print("Top Perimeter: " + topPerim + " Top Area: " + topArea);

    run("Make Inverse");       // invert selection & delete (gets rid of dirt spots etc..)
    setForegroundColor(255, 255, 255);
    run("Fill", "slice");
    run("Make Inverse");
    run("To Bounding Box");
    run("Crop");

    w = getWidth();  // image dimensions
    h = getHeight();
    topProfile = newArray(w);


for (x=0; x<w; x++){       // pixel column loop

    topProfile[x] = 0;

    for (y=0; y<h; y++){       // counts the number of white pixels in each column
```

```
		val = getPixel(x,y);
		if(val==255)
			topProfile[x] = topProfile[x] + 1;
	}
}


	topProfileCrop = newArray(0);

for(a=0; a<topProfile.length; a++){
	if(topProfile[a]>0){
		topProfileCrop = Array.concat(topProfileCrop, topProfile[a]);
	}
}

	topProfileMm = newArray(topProfileCrop.length);

for(a=0; a<topProfileCrop.length; a++){
	topProfileMm[a] = topProfileCrop[a] / topPixelsMm;		// make array of millimetre values
//	setResult("Top Pixels", a, topProfileCrop[a]);
//	setResult("Top Millimetres", a, topProfileMm[a]);
}

close();

// OPEN & PROCESS SIDE IMAGE

	open(sidePath);
	run("RGB Stack");
	run("Delete Slice");		// only uses the blue channel (channel with highest contrast)
	run("Delete Slice");
	//setTool(0);		//Rectangle tool
```

```
	//waitForUser("Select the Target", "Draw a box to cover the mouse ball\nensure no other dark objects are selected");

	//run("Crop");

	setThreshold(0, thresholdVal);
	run("Convert to Mask");

	medianScript = "radius="+smoothingVal+" slice";
	run("Median...", medianScript);	// threshold

	w = getWidth();	// image dimensions
	h = getHeight();

// SELECT CENTRE

	doWand(w/2, h/2, 1, "4-connected");

	List.setMeasurements;	// save measurements of perimeter & area
	sidePerim = List.getValue("Perim.");
	sideArea = List.getValue("Area");

	//print("Side Perimeter: " + sidePerim + " Side Area: " + sideArea);

	run("Make Inverse");	// invert selection & delete (gets rid of dirt spots etc..)
	setForegroundColor(255, 255, 255);
	run("Fill", "slice");
	run("Make Inverse");
	run("To Bounding Box");
	run("Crop");

	w = getWidth();	// image dimensions
```

```
		h = getHeight();
		sideProfile = newArray(w);

for (x=0; x<w; x++){		// pixel column loop

		sideProfile[x] = 0;

		for (y=0; y<h; y++){		// counts the number of white pixels in each column

			val = getPixel(x,y);
			if(val==255)
				sideProfile[x] = sideProfile[x] + 1;
		}
}


		sideProfileCrop = newArray(0);

for(a=0; a<sideProfile.length; a++){
		if(sideProfile[a]>0){
			sideProfileCrop = Array.concat(sideProfileCrop, sideProfile[a]);
		}
}

		sideProfileMm = newArray(sideProfileCrop.length);

for(a=0; a<sideProfileCrop.length; a++){
		sideProfileMm[a] = sideProfileCrop[a] / sidePixelsMm;		// make array of millimetre values
//		setResult("Side Pixels", a, sideProfileCrop[a]);
//		setResult("Side Millimetres", a, sideProfileMm[a]);
}
```

```
close();

// CIRCUMFRENCE CALCULATION

	arrayRatios = topProfileMm.length / sideProfileMm.length;

	circumfrenceArray = newArray(sideProfileMm.length);
	areaArray = newArray(sideProfileMm.length);

	for(i=0; i<sideProfileMm.length; i++){

		bLoc = round(arrayRatios*i);
		b = topProfileMm[bLoc]/2;
		a = sideProfileMm[i]/2;
		circumfrenceArray[i] = PI * (a+b) * (1+(3*pow((a-b)/(a+b),2) / (10+pow(4-(3*pow((a-
b)/(a+b),2)),0.5))));
		areaArray[i] = PI * a * b;
	}

	topLength =  topProfileMm.length / topPixelsMm;
	sideLength =  sideProfileMm.length / sidePixelsMm;
	aveLength = (topLength + sideLength)/2;
	mmPerSlice = aveLength/sideProfileMm.length; // average length of mouse

	VolumeSum = 0;
	AreaSum = 0;

for(a=0; a<sideProfileCrop.length; a++){	// loop to count the area and volume of each slice
	AreaSum = AreaSum + (circumfrenceArray[a]*mmPerSlice);
	VolumeSum = VolumeSum + (areaArray[a]*mmPerSlice);
//	setResult("Circumfrence", a, circumfrenceArray[a]);
```

```
}

EndArea = 0;

for(a=0; a<sideProfileCrop.length-1; a++)

EndArea = EndArea + (pow(pow(areaArray[a]-areaArray[a+1],2),0.5)); // sums end surface values - always positive

AreaSum = AreaSum + EndArea;

// SPHERICITY CALCULATION - this takes the average value of the area to circumference ratio of the top & side images

topCircleArea = PI*pow((topPerim/(2*PI)),2);        // this calculates the area a circle would have of the observed perimeter length

sideCircleArea = PI*pow((sidePerim/(2*PI)),2);

topRoundness =  topArea / topCircleArea;   // perfect circle area /observed area, so a perfect circle would be 1, going down to zero with worse roundness

sideRoundness = sideArea / sideCircleArea;

sphericity = (topRoundness+sideRoundness)/2;        // average roundness

// PRINT RESULTS

print("\n.............................................................");

print("Top-down image: " + topFileList[z]);

print("Side-on image: " + sideFileList[z]);

print("Sphericity:\t"+ sphericity);

print("Volume (mm^3):\t"+VolumeSum);

} //end batch processing loop
```
